# Using genome comparisons of wild-type and resistant mutants of *Methanococcus maripaludis* to help understand mechanisms of resistance to methane inhibitors

**DOI:** 10.1099/acmi.0.000244

**Published:** 2021-07-21

**Authors:** Feng Long, Chen-Yi Cheung, William B. Whitman, Gregory M. Cook, Ron S. Ronimus

**Affiliations:** ^1^​ Department of Microbiology, University of Georgia, Athens, Georgia, USA; ^2^​ Department of Microbiology and Immunology, University of Otago, Dunedin, New Zealand; ^3^​ Rumen Microbiology, AgResearch Ltd., Palmerston North, New Zealand; ^†^​ Present address: Department of Inflammation and Immunity, Cleveland Clinic, Cleveland, OH, USA

**Keywords:** chloroform, echinomycin, methanogen, methanogen-specific inhibitors, neomycin, rumen, whole-genome sequencing

## Abstract

Methane emissions from enteric fermentation in the ruminant digestive system generated by methanogenic archaea are a significant contributor to anthropogenic greenhouse gas emissions. Additionally, methane produced as an end-product of enteric fermentation is an energy loss from digested feed. To control the methane emissions from ruminants, extensive research in the last decades has been focused on developing viable enteric methane mitigation practices, particularly, using methanogen-specific inhibitors. We report here the utilization of two known inhibitors of methanogenic archaea, neomycin and chloroform, together with a recently identified inhibitor, echinomycin, to produce resistant mutants of *Methanococcus maripaludis* S2 and S0001. Whole-genome sequencing at high coverage (> 100-fold) was performed subsequently to investigate the potential targets of these inhibitors at the genomic level. Upon analysis of the whole-genome sequencing data, we identified mutations in a number of genetic loci pointing to potential mechanisms of inhibitor action and their underlying mechanisms of resistance.

## Introduction

Methane is a potent global greenhouse gas with a global-warming potential 28-fold higher than CO_2_ and whose concentration in the atmosphere has increased from less than 0.6 to 1.8 ppm during the last century [1, 2]. Enteric fermentation of feed by ruminants (cattle, sheep, goats) constitutes one of the main anthropogenic sources of atmospheric methane [3]. Ruminants emit about 100 million tons of methane per year, approximately 20 % of global methane emissions [4]. In the intestine of domestic ruminants, methane is produced by methanogenic archaea mainly using CO_2_ and H_2_, which then escapes into the atmosphere by eructation and breathing of the animals [1]. Moreover, the formation of methane reduces the feed efficacy of livestock, resulting in a loss of up to 12 % of the gross energy ingested by the animal [5]. Over the last decades, a number of potential enteric methane mitigation practices have been extensively studied [6–10], such as alternative forages [11], animal breeding [12], plant secondary products [13], control of protozoal populations [9, 14], vaccines [15] and alternative electron acceptors to divert H_2_ from methanogenesis and chemical inhibitors [6, 7, 16].

The use of chemical inhibitors is recognized as one of the most promising mitigation strategies for achieving large reductions in methane emissions [7]. Rumen methanogens are archaea and targeting their unique enzymes should lower the potential for off-target effects on the fibre-degrading bacteria, fungi and protozoa consortium or the ruminant host. The utilization of methanogen inhibitors as mitigation agents, particularly chemical compounds with inhibitory effects on methanogenic archaea, such as bromochloromethane, 2-bromoethane sulfonate, chloroform and cyclodextrin, have been extensively studied in various ruminant species recently [3, 17–20]. Other compounds known to inhibit the growth of methanogens are anthraquinones and various plant secondary compounds, such as garlic essential oils and allicin [7, 21, 22]. One major obstacle in the development of methanogen inhibitors for use in ruminants is that their effect can reduce overtime, sometimes completely [20] within a few days (e.g. with 2-bromoethane sulfonate;18). In a few cases, long-term *in vivo* experiments with inhibitors have reported up to 55–60 % inhibition [3, 17]. A recently described inhibitor in commercial development, 3-nitrooxypropanol, which targets the methane-producing enzyme methyl-coenzyme M reductase, is showing promise and inhibited the growth of methanogenic archaea without negatively affecting other non-methanogenic bacteria or the livestock [1, 23]. Despite the advances made to date, it remains unclear whether continuous use of inhibitors could slowly lead to resistance developing in the methanogens over time reducing the commercial utility of the inhibitors. Unfortunately, at present relatively little is known about how methanogens develop resistance to inhibitors at the genome level, which could limit our ability to develop inhibitors recalcitrant to resistance formation that will be required for mitigating greenhouse gas emissions over the coming decades. Due to the low cost of carbon, even relatively small reductions in effectiveness (two–threefold) resulting from small and unexpected changes in the genome could lead to setbacks in the use of an inhibitor on farms. In addition, the targets of some compounds remain unknown or unconfirmed and the specific molecular mechanisms that lead to resistance in methanogens to a particular compound remain unclear. In one recent report highlighting the unexpected nature of mutations that can arise due to the use of inhibitors, the antibiotic nourseothricin, which acts by causing translational miscoding of mRNA, was used to examine resistant mutants in four methanogen species and the mutant strains genome sequenced [24]. Four SNP mutations were found in resistant strains of *Methanobrevibacter smithii*, three of them in hypothetical proteins and one in a potassium transporter. In another study investigating cadmium resistance in *Methanosarcina acetivorans*, no genetic changes were found, suggesting that metabolic changes as sufficient to explain the observed resistance [25]. Despite the promising outlook for the use of inhibitors in controlling methane emissions, the effects of these compounds on animal health, food safety and environmental impact remain a significant concern.

Echinomycin, found in streptomycetes, is a cyclic octadepsipeptide antibiotic with two quinoxaline rings that bisintercalate into DNA [26]. Echinomycin is composed of two depsipeptides containing d-serine, l-*N*-methylvaline, l-*N*-methylcysteine and l-alanine with a quinoxaline base attached to d-serine [27]. The two peptide strands are joined by a thioacetal bridge and two ester linkages between d-serine and l-*N*-methylvaline [27]. Echinomycin has been shown to have activity against vancomycin-resistant enterococci [28], hypoxia-inducible factor-1 suppression [29], HIV-1 Tat transactivation inhibition [30], antithrombotic activity [31], and against methicillin-resistant *Staphylococus aureus* [32]. This antibiotic was recently identified as a growth inhibitor of *M. maripaludis* S2 in the low 2 µm concentration range [33].

Chloroform is a well-known potent inhibitor of methanogens [34]. The methane production in rumen fluid was inhibited 50 % within 79 min after the addition of 7.8 µm chloroform [34]. In addition, chloroform reduces rumen methane production to the same extent as bromochloromethane, however, with little or no negative influence on rumen fermentation in dairy cows and *in vitro* [20, 35]. Due to these advantages, chloroform together with other halogenated compounds, such as bromoethanesulfonate, have been used as experimental models in ruminant research in the last decades [10, 20, 35]. Chloroform has been proposed to interfere with the transfer of the methyl group from methyl-tetrahydroxymethanopterin (methyl-H_4_MPT) to coenzyme M (CoM), at the cobamide-dependent methyl-H_4_MPT:HS-CoM methyltransferase (Mtr) step of the methanogenesis pathway [36, 37]. However, the specific mechanism is not known yet.

Neomycin is an aminoglycoside antibiotic that inhibits both the growth of *M. maripaludis in vivo* and protein synthesis *in vitro* [38, 39]. The minimal inhibitory concentration of neomycin to *M. maripaludis* was previously determined to be 1.1 mm [39, 40]. Moreover, neomycin has already been utilized as a selectable marker in the development of genetic systems in *M. maripaludis* [40]. To deliver neomycin resistance in *M. maripaludis*, the aminoglycoside phosphotransferase genes APH3’I and APH3’II were cloned under the control of the *M. voltae* methyl reductase promoter and transformed into *M. maripaludis* [40].

In this report, we sought to investigate the underlying mechanisms of resistance to the methanogen inhibitors neomycin, chloroform and echinomycin in the genetically tractable model methanogen *Methanococcus maripaludis. Methanococcus maripaludis* is an excellent model organism to study methanogen metabolism and physiology due to its rapid and reliable growth, availability of a complete genome sequence and a set of well-developed genetic manipulation strategies [41, 42]. Spontaneous resistant mutants of echinomycin, chloroform or neomycin were produced from *M. maripaludis* wild-type strain S2 or a closely related variant S0001. These mutant strains, together with their parental strains, were subjected to whole-genome sequencing using Illumina sequencing. Overall, these mutational events may provide key insights into the resistant mechanisms of the tested methanogen inhibitors on the genome-wide scale.

## Methods

### Strains, media and growth conditions

Two *Methanococcus maripaludis* parental strains, the wild-type S2 and S0001, were used in this study. *M. maripaludis* S0001 is a mutant derived from wild-type strain S2 by the deletion of the gene encoding hypoxanthine phosphoribosyltransferase (MMP0145) and the addition of the gene encoding the *rep* gene from the *Methanococcus* shuttle vector pURB500 [43]. It is frequently used in a marker-less mutation system and as a host for shuttle vectors of *M. maripaludis* [43, 44]. Cultures were grown in McFAA medium (a formate minimal medium supplemented with 10 mm sodium acetate and 1 mm alanine) reduced with 3 mm dithiothreitol as indicated [45]. The 5 ml cultures were grown in 28 ml aluminium seal tubes pressurized to 103 kPa with N_2_/CO_2_ (4 : 1, v/v). The agar medium was prepared in 70 ml serum bottles and 10 ml of McFAA with 1 % (w/v) agar [45]. Serum bottles were pressurized to 103 kPa with N_2_/CO_2_ (4 : 1, v/v). Before inoculation, 3 mm sodium sulfide was added as the sulphur source. Echinomycin (> 98 % purity, CAS #: 512-64-1), chloroform (> 99 % purity) and neomycin (> 99 % purity) were purchased from Sigma-Aldrich (USA).

### Isolation of *M. maripaludis* echinomycin-resistant mutants from strains S2 and S0001

**Fig. 1.**
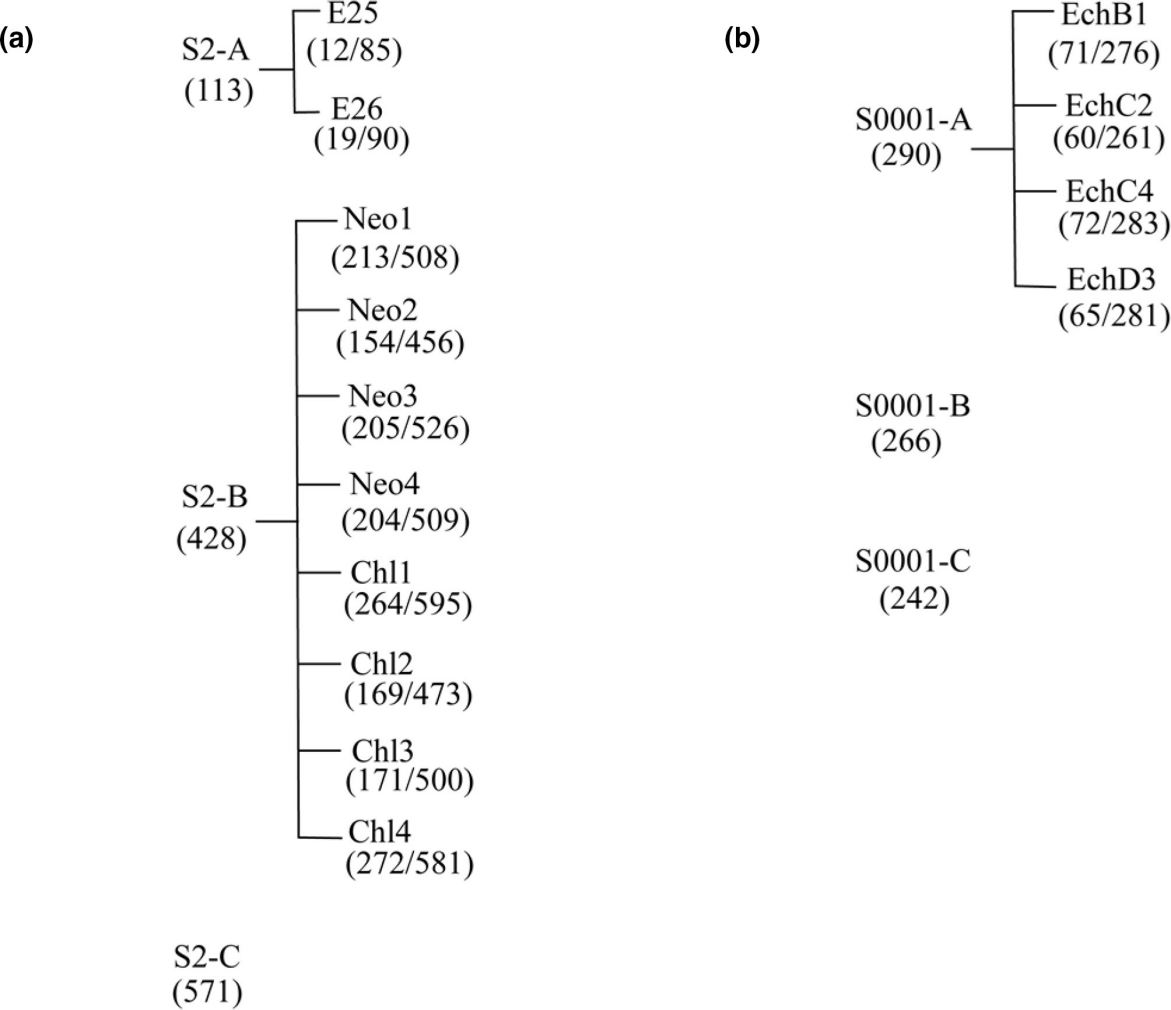
Strain tree describing the parental *M. maripaludis* wild-type strains and the corresponding derived resistant mutant strains used in this study. (a) *M. maripaludis* wild-type S2 strain A and its derived echinomycin-resistant mutants E25 and E26; *M. maripaludis* wild-type S2 strain B and its derived neomycin resistant mutants Neo1, Neo2, Neo3 and Neo4, together with its derived chloroform resistant mutants Chl1, Chl2, Chl3 and Chl4; *M. maripaludis* wildtype S2 strain C is a sub-strain of wild-type S2 strain B. (b) *M. maripaludis* wild-type S0001 strain A and its derived echinomycin-resistant mutants Ech1, Ech2, Ech3 and Ech4; *M. maripaludis* wild-type S0001 strain B and C are subculture strains of wild-type S0001 strain A. Numbers under the wild-type strains represents the number of SNPs found in the parental strain when compared to the S2 reference genome. The first number under the resistant mutant strains is the number of SNPs found only in the mutant but not in its parental strain, while the second number is the total number of SNPs found in the mutant strain. S2 reference genome [21] was used for comparison for both results.

**Fig. 2.**
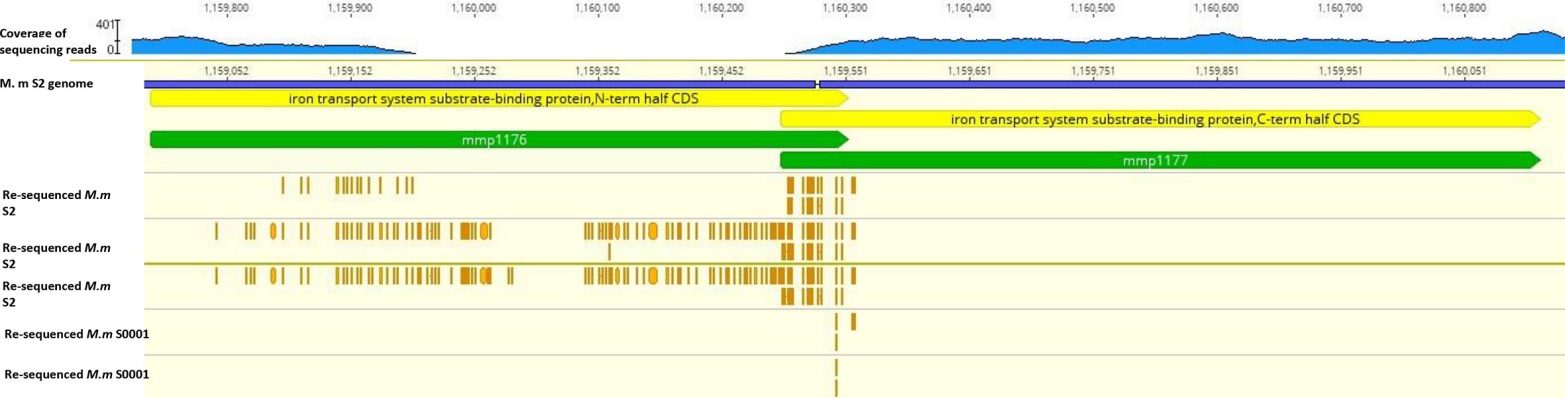
Partial genomic map of the region position 1 158 991 to 1 160 125 presenting variant SNPs of re-sequenced *Methanococcus maripaludis* wild-type strains S2-A, S2-B, S2-C, S0001-A and S0001-B mapped back to the S2 reference genome. Blue rectangles represent the positions 1 158 991 to 1 160 125 of *M. maripaludis* S2 genome [53]. Green bars stand for the genes in this region on *M. maripaludis* S2 genome. Orange bars represent variant SNPs of each strain, they were 13, 73, 31, 1, 1, 1 variant SNPs on MMP1176 from top to bottom, with 88.9−99.1 %, 81.8−99.8 %, 88.1−100 %, 14.7, 19.8, 10  % variant frequency, respectively; and 7, 12, 12, 2, 1,2 variant SNPs on MMP1177 from top to bottom, with 89.5−98.6 %, 99.1−99.8 %, 99.5−100 %, 14.70, 71.4–75.8 %, 19.8, 10, 72.3–74.8 % variant frequency, respectively.

Echinomycin-resistant mutants were generated in strains S2 and S0001 using a serial culture method. These two strains were named as S2-A and S0001-A, respectively in this study (Fig. 1). For these experiments, an inoculum of 1.5×10^5^ cells of strain S2 was challenged with 0.1, 0.2, 0.5, 1, 2 and 5 µm of echinomycin in McFAA medium. Three replicate cultures were used for each concentration. To obtain individual echinomycin-resistant mutants, three different inoculum sizes of wild-type *M. maripaludis* S2 (1.5×10^5^, 1.5×10^6^ and 1.5×10^7^ cells) were plated onto defined agar medium with three concentrations of echinomycin (0.5, 1 and 2 µm) for nine conditions in total. Isolated colonies were only observed on agar plates with 0.5 µm echinomycin at a frequency of about one in 10^5^. To screen isolated resistant mutants, 20 colonies were picked from these agar cultures and inoculated into McFAA medium containing 1 µm echinomycin. Among these 20-independent resistant mutant lines, six lines were chosen for next-step experiments. These six parallel mutant lines were then propagated in 5 ml McFAA medium with 1.0 µm echinomycin. Cultures were transferred every 24 h by inoculating ~5 % (v/v) of the culture to 5 ml fresh medium, four times in total. The absorbance of these six mutant lines during each transfer was recorded. Two lines, Ech 25 and Ech 26, with the highest growth rate, were selected for whole-genome sequencing. After the selection of resistance mutants was finished, six echinomycin-resistant mutants, including two S2-derived mutants and four S0001-derived mutants, were subjected to whole-genome sequencing using Illumina technology (Fig. 2).

In parallel, echinomycin-resistant mutants were isolated from strain S0001-A. *M. maripaludis* S0001 was retrieved in McFAA medium with 2 % (w/v) Casamino acids from a glycerol stock and saved as the parental strain for later resequencing. Three parallel broth cultures, EchB, EchC, and EchD were inoculated with 10^6^ cells from this seed culture and grown to early stationary phase. Cells, 10^6^, from each lineage were inoculated into defined medium agar bottles with 0.5 µm echinomycin. Twelve colonies were picked from these agar bottles (four colonies from each lineage) and inoculated into McFAA medium containing 0.5 µm echinomycin for further characterization. These 12-parallel mutant lines were then subcultured in 5 ml McFAA medium with 0.5 µm echinomycin by inoculating 10^5^ cells. The absorbance of these 12 mutant lines during each transfer was recorded. Three mutant lines, Ech B1, Ech C4 and Ech D3 that had the highest growth rate, together with mutant Ech C2 that had the lowest growth rate, were selected for whole-genome sequencing.

### Isolation of chloroform and neomycin resistance mutants

When isolating resistant mutants, an inoculum of 1.5×10^5^ cells of *M. maripaludis* S2 cells was challenged with 5, 10 and 20 µm of chloroform, or 100, 169, 338, and 677 µm of neomycin in McFAA broth medium [46]. This parental strain was named as S2-B (Fig. 2). Replicate cultures were made for each line. Then, 10 % (v/v) of stationary phase cultures from McFAA broth medium with 10 µm chloroform or 169 µm neomycin from the previous step were plated to McFAA agar medium in the presence of 10 um chloroform or 100 um neomycin, respectively. Replicates were made for each line. The following steps of propagating the resistant mutations were performed in the anaerobic chamber containing a gas mixture of 4 % H_2_, 5 % CO_2_ and 91 % N_2_. Forty colonies of chloroform- or neomycin-resistant mutants were picked and inoculated into individual wells of a 96-well microtitre plate. Each well contained 320 µl McFAA broth medium with 10 µm of chloroform or 100 µm of neomycin. Resistant cultures (15 in total) from each antibiotic were pooled together and further transferred three times in 28 ml aluminium seal tubes. Mutant cells were transferred every 24 h by inoculating ~10 % (v/v) of the culture into 5 ml McFAA broth medium with either chloroform or neomycin. Starting with 10 µm of chloroform or 100 µm of neomycin, the dosage was increased to 15 µm of chloroform or 150 µm of neomycin at the second transfer, and to 20 µm of chloroform or 200 µm of neomycin at the third transfer. Four chloroform-resistant mutants and four neomycin-resistant mutants from the last transfer were selected for whole-genome sequencing (Fig. 1).

### Whole-genome sequencing of wild-type and resistant mutants

Genomic DNA from the resistant mutants and the corresponding parental strains were isolated using Quick-DNA Fungal/Bacterial Miniprep Kit from Zymo Research (CAS No.: D6005) or Qiagen DNeasy Blood and Tissue Kit. For the six echinomycin-resistant mutants and their parental strains, 1 µg of genomic DNA for each strain were sheared into 350 bp fragments by the Covaris E220 Evolution instrument at the Georgia Genomics facility. The genomic DNA library for sequencing was then constructed using NEBNext Ultra DNA Library Prep Kit for Illumina (CAS No.: E7370). These eight genomic libraries were sequenced by Illumina NextSeq Paired-End sequencing with 25–30 Gb bases being read, operated by the Georgia Genomics facility. The four chloroform-resistant mutants, four neomycin-resistant mutants and their parental strain S2 was sequenced using paired-end 250 bp reads on an Illumina MiSeq (Illumina, Hayward, CA).

### Bioinformatics analysis of genome sequences

Sequencing data were analysed using Geneious 10 software [46]. The pair-ended readings of each strain were first mapped back to the *M. maripaludis* S2 genome sequence [42]. To look for variant SNPs of each strain, the SNPs were called with the variant frequency of at least 0.1 and a 10^−6^ maximum variant *P*-value. By using the ‘Find Variant SNP’ function in Geneious 10, the SNPs of each resistant mutant was subtracted from that of their corresponding parental strains, SNPs unique to each resistant mutant were then collected. To exclude those mutations that existed before the antibiotic selection began and sequencing artefacts, these variant SNPs of resistant mutants were manually examined to remove any mutations that also existed in the parental strains. Variant polymorphisms present in echinomycin-, chloroform- or neomycin-resistant mutants were then identified. Gene synteny of some identified proteins was examined using SYNtax [47] and blast searches from NCBI database [48]. The total number of SNPs of the resistant mutants and the number of variant SNPs from their parental strains were summarized in Table S2. The NCBI accession numbers for the 20 genome sequences are SRX10350745–SRX10350764 and have been assigned the overarching NCBI SRA database project code PRJNA714766.

## Results

### Whole-genome sequencing of *M. maripaludis* echinomycin-resistant mutants

The MIC of echinomycin to *M. maripaludis* was first determined in broth culture and was found to be 1 µm. Growth was not observed in any of the tubes when the concentration of echinomycin was > 1.0 µm echinomycin. At concentrations < 0.5 µm echinomycin, growth was observed in some but not all of the replicate tubes, which was taken as evidence for selection of spontaneous resistance mutants [49]. Isolated colonies were observed at a frequency of about one in 10^4^. Because *M. maripaludis* is polypoid and contains 20–50 copies of the genome per cell [45], it was possible that resistance could have resulted from dominant mutations in a small fraction of the alleles. For that reason, all mutations that occurred in at least 10 % of the sequencing reads were examined.

While numerous mutations were detected in echinomycin-resistant mutants, only two loci contained shared mutations in S2-derived mutants (File S1, available in the online version of this article). These two loci were present in a 580 bp intergenic region between *mmp0478* and *mmp0479*. These two genes encode proteins of unknown function. For the S0001-derived mutants, no mutations were identified that were shared by all four mutants (File S2). Ten mutations were shared in two or three of these S0001-derived mutants. However, none of these mutational events seemed to be functionally relevant to echinomycin resistance.

### Whole-genome sequencing of *M. maripaludis* chloroform- and neomycin-resistant mutants

The MIC of chloroform and neomycin to *M. maripaludis* S2 was first determined in broth culture and found to be 1 and 68 µm, respectively. In tubes with the highest concentration of chloroform or neomycin, growth was not observed. Growth was observed in some of the tubes with the lower concentrations of chloroform or neomycin. In the chloroform-resistant mutants, six shared mutations, with at least 86 % frequency, were identified (Table 1, File S3). They occurred in two intergenic regions of the *M. maripaludis* genome, in *mmp_rs07920* (tRNA-IIe), and in three protein coding genes *mmp007* (geranylgeranylglyceryl phosphate synthase), *mmp1115* (transketolase) and *mmp1689* (ComE). Interestingly, these six mutations were also found in the neomycin-resistant mutants (Tables 1 and 2, File S4). The mutations that occurred at the first five loci were identical in both two types of resistant mutants, suggesting that they were derived from the parental strain. However, in *mmp1689* (encoding ComE), the mutations were different. First, a deletion of nucleotide T at position 1 630 332 was found in both chloroform-resistant mutants and neomycin-resistant mutants. Second, another three mutational events, an insertion and two transversions, were found in the nearby positions in neomycin-resistant mutants only. In the neomycin-resistant mutants, a deletion in *mmp0535* (hypothetical protein) and a transversion in *mmp0707* (sodium: proton antiporter) were also found in all neomycin-resistant mutants (Table 2).

**Table 1.** Variant SNPs found in three or four of the chloroform-resistant mutants

SNPs	Encoded protein/tRNA	Variant frequency	Polymorphism type	Amino acid change	Protein effect
Non-coding region at 107 992*	n/a	92.1 % to 100 %	Transversion	n/a	n/a
Non-coding region at 1 588 772*	n/a	91.1 % to 100 %	Transition	n/a	n/a
MMP_RS07920*	tRNA-IIe	91.0 %t o 100 %	Transition	n/a	n/a
MMP0007*	Geranylgeranylglyceryl phosphate synthase	89.3 % to 99.9 %	Transversion	L → H	Substitution
*MMP1115* *†	Transketolase	88.2 % to 99.8 %	Transition	L → P	Substitution, frame shift
MMP1689*	ComE	86.2 % to 100 %	Deletion	n/a	Frame shift, substitution

n/a, not applicable.

*SNPs also found in neomycin-resistant mutants.

†SNPs shared by three out of four chloroform-resistant mutants. The rest of the SNPs were found in all four chloroform-resistant mutants.

**Table 2.** Variant SNPs found in at least three out of four neomycin-resistant mutants

SNPs	Encoded protein/tRNA	Variant frequency	Polymorphism type	Amino acid change	Protein effect
Non-coding region at 107 992*	n/a	100 %	Transversion	n/a	n/a
Non-coding region at 1 588 772*	n/a	99.3 % to 100 %	Transition	n/a	n/a
MMP_RS07920*	tRNA-IIe	98.4 % to 100 %	Transition	n/a	n/a
mmp0007*	Geranylgeranylglyceryl phosphate synthase	99.6 % to 99.90 %	Transversion	L→H	Substitution
*mmp0535*	Hypothetical protein	100 %	Deletion	n/a	Frame shift
*mmp070*†	Sodium:proton antiporter	11.6 % to 99.5 %	Transversion Transition	G → C, A → V	Substitution
mmp1115*	Transketolase	99.8 % to 99.90 %	Transition	L → P	Substitution
mmp1689*	ComE	11.4 % to 100 %	Insertion, Transversion Deletion	S → T	Frame shift, substitution

n/a, not applicable.

*SNPs also found in chloroform-resistant mutants.

†SNPs shared by three out of four neomycin-resistant mutants. The remaining SNPs were found in all four neomycin-resistant mutants.

### Mutations in re-sequenced *M. maripaludis* S2 and S0001 strains

Three *M. maripaludis* wild-type S2 strains and three S0001 strains were re-sequenced in this experiment (Fig. 1). They were a *M. maripaludis* S2 strain that was the parental strain of neomycin- and chloroform-resistant mutants, S2-B; a subculture of this S2 parental strain, S2-C; another S2 strain that was the parental strain for generating echinomycin-resistant mutants, S2-A; a *M. maripaludis* S0001 strain that was the parental strain for generating echinomycin-resistant mutants, S0001-A; and two S0001 sub-cultures of this parental strain, S0001-B and S0001-C. To look for mutations that existed in all re-sequenced *M. maripaludis* wild-type strains, variant SNPs with at least 80% of frequency were collected and compared (Tables 3 and 4). Several mutations that existed in all three re-sequenced S2 genomes were identified, and they all occurred at a frequency of least 89 % (Table 3). Strikingly, a variety of mutational events, such as transitions, transversions, insertions and substitutions, were found in *mmp1176* and *mmp1177,* with a frequency of 88.9 % to 100 % (Fig. 2). Similar but fewer mutations, with a frequency of 10% to 75.8 %, were also mapped in these two genes in the three re-sequenced S0001 strains (Table 4). The emergence of these mutations in *mmp1176* and *mmp1177* may have reduced the activities of the encoded proteins. *mmp1176* and *mmp1177* are overlapping genes that encode the N-terminal and C-terminal regions of a substrate-binding protein of an iron transport system, respectively. One tentative interpretation of this observation is that mutations in the laboratory strains of *M. maripaludis* may have accumulated following extended cultivation in medium where iron was readily available. The iron concentration for cultivating *M. maripaludis* in the laboratory is usually around 25 µm [40], a concentration close to that of this organism’s original habitat in marine sediments [50, 51]. However, the real available iron for *M. maripaludis* in their native environment is unknown. In laboratory cultivated *M. maripaludis*, iron was constantly provided in medium. Therefore, it was possible that the accumulation of mutations in iron-transport protein MMP1176 and MMP1177 was driven by the selection towards less competition for iron than in the organism’s original habitat. Another two mutations found in all re-sequenced S2 and S0001 strains were on *mmp1477* and *mmp1478*. These two genes together encode a cobyrinic acid a,c-diamide synthase. The last mutation that has been found on both re-sequenced S2 and S0001 strains was an A nucleotide deletion on the position of 1 495 465 in an intergenic region. This region is between the gene annotated as encoding cytochrome c haem-binding site (*mmp1537*) and a gene of unknown function (*mmp1536*). As these mutations were identical in all S2 and S0001 strains, they were unlikely to be involved in the acquisition of resistance.

**Table 3.** Variant SNPs, with at least 80 % frequency, found in three re-sequenced *M. maripaludis* S2 strains

SNPs	Encoded protein/tRNA	Variant frequency	Polymorphism type	Amino acid change	Protein change
Intergenic region at 1 495 465*	n/a	99.4 % to 99.9 %	Deletion	n/a	**n/a**
**Intergenic region at 454 886**	n/a	98.1 % to 100 %	Substitution	n/a	**n/a**
***mmp0253***	CoA-binding domain-containing protein	99.8 % to 100 %	Transition	G→E	**Substitution**
***mmp0394***	Uroporphyrinogen III synthase	98.7 % to 99.8 %	Transition	D → N	**Substitution**
mmp1176*	Iron transport system substrate-binding protein, N-term half	88.9 % to 100 %	Transition, transversion, insertion, substitution	N →D, G→ D, E → Q ^……*^	**Substitution, truncation,** **frame shift**
mmp1177*	Iron transport system substrate-binding protein, C-term half	89.5 % to 100 %	Transition, transversion, insertion, substitution	E→N, L→S, F→T, ^……*^	**Substitution, truncation,** **frame shift**
***mmp1466***	CBS domain-containing signal transduction protein	99.4 % to 100 %	Transition	D→G	**Substitution**
***mmp1477***	Cobyrinic acid a,c-diamide synthase	99.3 % to 100 %	Deletion	n/a	**Frame shift**
***mmp1478***	Cobyrinic acid a,c-diamide synthase:cobyrinic acid a,c-diamide synthase CbiA	99.3 % to 100 %	Deletion	n/a	**Frame shift**

*Multiple mutations were identified, in total 86 variant SNPs were found in *mmp1176*, 19 variant SNPs were found in *mmp1177*.

**Table 4.** Variant SNPs found at a high frequency in three re-sequenced *M. maripaludis* S0001 cultures*

SNPs	Encoded Protein/tRNA	Variant frequency	Polymorphism type	Amino acid change	Protein change
**Intergenic region at 1 495 465**	n/a	99.4 % to 100 %	Deletion	n/a	**n/a**
***mmp0041***	Transcription initiation factor IIB	97.2 % to 100 %	Substitution	V → I	**Substitution**
***mmp0166***	MATE family drug/sodium antiporter	98.3 % to 99.2 %	Transversion	N → I	**Substitution**
***mmp0234***	Hypothetical protein	99.2 % to 100 %	Deletion	n/a	**Frame shift**
***mmp0697***	leucyl-tRNA synthetase	97.7 % to 100 %	Transition	I → V	**Substitution**
***mmp1013***	Carbamoyl-phosphate synthase	98 % to 99 %	Transversion	G → C	**Substitution**
***mmp1051***	Stationary phase survival protein SurE	98.8% to 100%	Deletion	N/A.	**Frame shift**
***mmp1169***	SufBD protein	98.3% to 100%	Insertion	n/a	**Frame shift**
mmp1176*	Iron transport system substrate-binding protein, N-term half	14.7% to 19.8%	Transversion	L → R	**Substitution**
mmp1177*	Iron transport system substrate-binding protein, C-term half	14.7% to 75.8%	Transversion	E → N	**Substitution**
***mmp1181***	Iron transport system binding protein	83.9% to 87.2%	Transition	D → G	**Substitution**
***mmp1305***	Hypothetical protein	98.1% to 100%	Transition	A →T	**Substitution**
***mmp1477***	Cobyrinic acid a,c-diamide synthase	98.8% to 100%	Deletion	n/a	**Frame shift**
***mmp1478***	Cobyrinic acid a,c-diamide synthase:cobyrinic acid a,c-diamide synthase CbiA	98.8% to 100%	Deletion	n/a	**Frame shift**
***mmp1593***	Hypothetical protein	97.3% to 99.1%	Transversion	G → V	**Substitution**
***mmp1624***	Polyferredoxin	98.6 % to 99.3 %	Insertion	n/a	**Frame shift**

*All variant SNPs summarized here occurred at a frequency of at least 80 %, except in *mmp1177* and *mmp1176*.

Other mutations found only in the re-sequenced S2 strains were the following: a nucleotide C to T transition on the position of 255 618 from *mmp0253*, encoding a hypothetical protein. This mutation altered the original amino acid glycine into glutamic acid. Two, a nucleotide changed from C to T on the position of 392 113 of *mmp0394*, encoding uroporphyrinogen III synthase. This mutation changed an amino acid from aspartic acid to asparagine. Lastly, a nucleotide changed from A to G on the position of 1 428 254 from *mmp1466*, encoding a CBS domain-containing signal transduction protein. This mutation changed an amino acid from aspartic acid to a glycine. Because these three mutation events were not independent among all re-sequenced S2 strains, possibly, they were structural variants without any functional disruptions.

In the re-sequenced *M. maripaludis* S0001, both *mmp0145* (hypoxanthine phosphoribosyltransferase) and *mmp0680* (uracil phosphoribosyltransferase) were deleted during the construction of this strain from strain S2, as confirmed in our re-sequencing data (Table 4). Other polymorphisms, all identical in these three S0001 strains, are summarized in Table 4.

## Discussion

We present here a genome-sequence-based analysis of *M. maripaludis* S2 strains resistant to three inhibitors, echinomycin, chloroform or neomycin, attempting to explore the resistance mechanisms on a genome-wide scale. Mutant strains resistant to echinomycin, chloroform or neomycin were first generated by serial transfers in the presence of sub-lethal levels of the inhibitors. Based on the subsequent whole-genome sequencing analysis, a number of SNPs were identified in all resistant mutants.

### Echinomycin

In this study, only two unique, shared mutations were identified in S2-derived echinomycin-resistant mutants. These two mutations mapped to an intergenic region between the genes *mmp0478* and *mmp0479* (conserved hypothetical proteins with no annotated function) suggesting an effect on gene transcription. No shared mutations were found in the S0001-derived resistant mutants. These observations suggested that resistance may have resulted from multiple mutational events. Alternatively, mutations may be dominant and never become abundant in the cells of these polyploid micro-organisms [52]. When producing the resistant mutants, the *M. maripaludis* S2 or S0001 strains were challenged with 0.5 µm echinomycin, which is below the MIC of this compound (1 µm). Isolated S2-derived resistant mutants from this step were further transferred four times in the presence of 1 µm echinomycin. These four passages propagated up to ~17 generations. At the same time, S0001-derived resistant mutants were further transferred once in the presence of 0.5 µm echinomycin. That is, up to ~13 generations were propagated. Similarly, up to ~10 generations were propagated for chloroform or neomycin-resistant mutants. A former study of antibiotic-resistant mechanisms with *E. coli* resistant mutants used up to 61 independent lines with up to ~336 generations of propagation [53]. A total of 402 independent mutational events were detected in all *E. coli* resistant mutants [53]. Therefore, in future experiments of preparing resistant mutants to study the resistant mechanisms at the genomic level, refinements such as applying a higher dose of inhibitor and increasing the strength of propagating the mutation, should be considered.

Several resistance mechanisms in micro-organisms that produce echinomycin organisms (such as *Streptomycetes*) have been proposed including DNA repair, the utilisation of an ATP-dependent permease or the sequestration of the compound by binding to a cell protein [54]. It is unclear at present the function of the encoded proteins by genes *mmp0478* and *mmp0479* and how they are involved in aiding resistance to echinomycin. Further studies are required, which could use gene-knockout strains generated by transposon mutagenesis [55].

### Neomycin and chloroform

Bacterial resistance to the aminoglycoside antibiotics, such as neomycin, is mostly associated with the expression of modifying enzymes which phosphorylate, adenylate or acetylate these compounds [56]. Three types of aminoglycoside-modifying enzymes were reported so far, they are *O*-phosphotransferase (APHs), *O*-adenyltranferases (ANTs) and *N*-acetyltransferases (AACs) [56]. However, none of these protein homologs exist in *M. maripaludis*. Other resistance mechanisms, such as diminished cell-membrane permeability, structural alteration in the ribosomal target of the antibiotic, or extrusion of the aminoglycosides from the cell by efflux pumps, have also been proposed [57]. In the methanogen *Methanothermobacter thermautotrophicus* strain ΔH, which is phylogenetically related to the dominant methanogens in the rumen, resistance to neomcyin was indicated to be associated with mutation(s) in a crucial energy producing sodium translocating ATPase [58].

In our study, two shared mutations were identified only in neomycin-resistant mutants: [1] an unannotated protein (MMP0535); and [2], a sodium: proton antiporter (MMP0707), which is very interesting as this protein is critical for maintaining pH homeostasis. It is interesting that a change was observed in the sodium: proton antiporter whose function presumably shares overlap with that of the sodium-dependent ATPase in *M. thermautotrophicus* [58]. Three mutations in the protein-coding regions were detected in both neomycin- and chloroform-resistant mutants. These included geranylgeranylglyceryl phosphate synthase (MMP0007), transketolase (MMP1115) and ComE (MMP1689). These enzymes play critical roles in membrane biosynthesis, carbohydrate metabolism and cofactor biosynthesis. Changes in geranylgeranylglyceryl phosphate synthase could potentially have either led to changes in membrane composition affecting transport of the inhibitor or improved growth characteristics of the cells. Nonetheless, confirmation that these proteins are associated with neomycin and or chloroform resistance in *M. maripaludis* requires further genetic and biochemical validation.

In the study by Knight *et al*. [20], chloroform was examined as an experimental compound to examine the effects of an inhibitor in sheep over a 42 day dosing period. Initial reductions of approximately 90 % were obtained in the first week, but these reduced over time reduction at 42 days to 38 %. One would expect that long-term exposure to chloroform could lead to mutations in the MtrA encoding gene or other genes encoding other proteins that it interacts with in the MtrA-H complex [59, 60]. What this study suggests is that a significant level of resistance to an inhibitor can develop in unexpected ways and does not necessarily require specific mutations in the actual for target enzyme of the inhibitor.

By combining the generation of resistant mutants using serial transfer techniques with inhibitors under sub-lethal concentrations, together with the subsequent whole-genome sequencing analysis, this work has mapped a viable pipeline for exploring the targets of inhibitors and the underlying mechanisms of their resistance. The study identified a number of unexpected changes in genetic loci, highlighting that a significant level of resistance can arise through mutations that are not necessarily associated with the known target of an inhibitor. Although certain experimental details still require further refinements, such as increasing the propagation of the mutants (perhaps combined with higher concentrations of inhibitor), mutations that potentially associated with the inhibitor-resistant mechanisms were identified. This is a critical step to develop novel methanogen inhibitors, after a list of candidates has been collected from phenotypic screening techniques. Ultimately, the goal is to elucidate the underlying molecular mechanisms of resistance, which would be aided by the acquisition of the target enzyme structural information and any interactions with the inhibitors.

## Supplementary Data

Supplementary material 1
